# Effects of hepatitis C virus core protein and nonstructural protein 4B on the Wnt/β-catenin pathway

**DOI:** 10.1186/s12866-017-1032-4

**Published:** 2017-05-25

**Authors:** Xiao-Hua Jiang, Yu-Tao Xie, Ya-Ping Cai, Jing Ren, Tao Ma

**Affiliations:** 1 Department of Infectious Diseases, the First Affiliated Hospital of the University of South China, Hengyang, 421001 China; 2 Department of Infectious Diseases, Xiangya Hospital of Central South University, Changsha, 410087 China; 3 Department of Epidemiology and Health Statistics, the University of South China, Hengyang, 421001 China

**Keywords:** HCV, Core protein, NS4B, Wnt/β-catenin signaling pathway

## Abstract

**Background:**

Hepatitis C virus (HCV) core protein and nonstructural protein 4B (NS4B) are potentially oncogenic. Aberrant activation of the Wnt/β-catenin signaling pathway is closely associated with hepatocarcinogenesis. We investigated the effects of HCV type 1b core protein and NS4B on Wnt/β-catenin signaling in various liver cells, and explored the molecular mechanism underlying HCV-related hepatocarcinogenesis.

**Results:**

Compared with the empty vector control, HCV core protein and NS4B demonstrated the following characteristics in the Huh7 cells: significantly enhanced β-catenin/Tcf-dependent transcriptional activity (*F* = 40.87, *P* < 0.01); increased nuclear translocation of β-catenin (*F* = 165.26, *P* < 0.01); upregulated nuclear β-catenin, cytoplasmic β-catenin, Wnt1, c-myc, and cyclin D1 protein expression (*P* < 0.01); and promoted proliferation of Huh7 cells (*P* < 0.01 or *P* < 0.05). Neither protein enhanced β-catenin/Tcf-dependent transcriptional activity in the LO2 cells (*F* = 0.65, *P* > 0.05), but they did significantly enhance Wnt3a-induced β-catenin/Tcf-dependent transcriptional activity (*F* = 64.25, *P* < 0.01), and promoted the nuclear translocation of β-catenin (*F* = 66.54, *P* < 0.01) and the Wnt3a-induced proliferation of LO2 cells (*P* < 0.01 or *P* < 0.05). Moreover, activation of the Wnt/β-catenin signaling pathway was greater with the core protein than with NS4B (*P* < 0.01 or *P* < 0.05).

**Conclusions:**

HCV core protein and NS4B directly activate the Wnt/β-catenin signaling pathway in Huh7 cells and LO2 cells induced by Wnt3a. These data suggest that HCV core protein and NS4B contribute to HCV-associated hepatocellular carcinogenesis.

**Electronic supplementary material:**

The online version of this article (doi:10.1186/s12866-017-1032-4) contains supplementary material, which is available to authorized users.

## Background

Hepatitis C virus (HCV) is one of the main pathogens associated with hepatocellular carcinoma (HCC). It is estimated that approximately 185 million people worldwide have been infected with HCV, and more than 35 million people die annually from HCV-related liver diseases, the main diseases being cirrhosis and hepatocellular carcinoma [1]. However, the mechanisms underlying HCV-induced HCC have not been fully elucidated.

The HCV core protein comprises 191 amino acids, has RNA-binding activity, and forms the viral nucleocapsid. HCV core protein is necessary for the assembly and production of infectious virions, and is also involved in cell signaling, transcriptional regulation, apoptosis, lipid metabolism, and cell transformation [2, 3]. Furthermore, the core protein induce HCC in transgenic mice [4]. HCV nonstructural protein 4B (NS4B) is an integral membrane protein that comprises 261 amino acids. Electron microscopy, and biochemical, structural, and genetic studies have shown that NS4B is the key organizer in the formation of HCV replication complexes [5, 6]. NS4B induces the formation of a membranous web (MW), which is believed to provide support for HCV RNA replication [7, 8]. Moreover, NS4B has NTPase activity [9] and can bind to viral RNA [7]. However, the role of HCV NS4B in the occurrence of liver cancer remains unclear. Wang et al. [10] reported that transgenic mice with intrahepatic expression of NS4B do not develop tumors, but Einav et al. [11] found that the HCV NS4B gene mediates cell transformation and tumor formation.

The Wnt/β-catenin signaling pathway has been associated with tumor cell growth, metastasis, and recurrence, and its excessive activation is closely associated with the occurrence of HCC [12, 13]. Therefore, an investigation is warranted to determine whether HCV core protein and NS4B activate the Wnt/β-catenin signaling pathway, thereby inducing HCV-related HCC. Fukutomi et al. [14] reported that the HCV core protein upregulates Wnt1 and WISP2 in Huh7 cells, thereby promoting cell proliferation and cell cycle progression. Liu et al. [15] reported that in Huh7 cells, HCV core protein enhances Wnt3a-induced β-catenin/Tcf-dependent transcriptional activity via inactivation of glycogen synthase kinase-3β (GSK-3β) activity, thereby promoting the proliferation of Huh7 cells. However, these HCV studies were most often carried out in liver cancer cell lines [16], and only rarely in normal human liver cell lines. Research on the impact of NS4B on the Wnt/β-catenin pathway is also scarce. In our previous studies, we used reverse transcription–polymerase chain reaction (RT-PCR) to amplify the HCV core protein and NS4B genes from the sera of patients with chronic hepatitis C genotype 1b [17], and then fused them with the mkate2 gene, which expresses red fluorescent protein. Later, these genes were inserted into the lentiviral expression vector pLenti6.3/V5. This was followed by viral packaging and concentration, and inoculation into Huh7 and LO2 cells. Therefore, we successfully constructed human HCC Huh7 and normal human liver cell line LO2 models that stably expressed HCV core protein and NS4B. Based on this development, in the present study we explored the effects of HCV type 1b core protein and NS4B on the Wnt/β-catenin signaling pathway in Huh7 and LO2 cells, and examined the roles of those two proteins in the formation of HCV-related HCC. The results of this study will contribute to the formulation of new strategies to prevent and treat HCV-related HCC.

## Methods

### Cell culture

The human normal liver cell lines LO2 (American Type Culture Collection, Manassas, VA, USA) were cultured in 10% fetal bovine serum (FBS)-containing Roswell Park Memorial Institute (RPMI) 1640 medium (Gibco, Carlsbad, CA, USA) at 37 °C, 5% CO2, and saturated humidity. The human HCC lines Huh7 (American Type Culture Collection, Manassas, VA) were cultured in 10% FBS-containing Dulbecco’s modified Eagle’s medium (DMEM) (Gibco, Carlsbad, CA) at 37 °C, 5% CO_2_, and saturated humidity. The 293FT cell lines (Invitrogen, Carlsbad, CA, USA) were cultured in 10% FBS-containing DMEM supplemented with 0.1 mM non-essential amino acids, 1 mM sodium pyruvate and 2 mM L-glutamine (Gibco, Carlsbad, CA) at 37 °C, 5% CO^2^, and saturated humidity.

### Construction of lentiviral vectors expressing HCV Core, NS4B and mkate2

To improve the expressions of HCV Core and NS4B genes in Huh7 and LO2 cells, we used a lentiviral expression system (Invitrogen, Carlsbad, CA). In brief, HCV Core and NS4B genes were amplified from plasmids of pDsRed-monomer-Core and pDsRed-monomer-NS4B ([17], constructed in our lab), and then fused them with mkate2 gene, which was amplified from pmkate2-N plasmid (Invitrogen, Carlsbad, CA) by polymerase chain reaction (PCR) using primers listed in Additional file 1: Table S1. Subsequently, these genes were cloned into the pLenti6.3/V5 expression vector (Invitrogen, Carlsbad, CA) by BamHI and AscI digestion and T4 DNA ligase ligation, generating the three pLenti6.3-based vectors expressing HCV Core, NS4B and mkate2: pLenti6.3-Core, pLenti6.3-NS4B and pLenti6.3-mkate2. The inserted DNA fragments were verified by sequencing.

### Establishment of stable cell lines expressing HCV Core protein, NS4B and mkate2 protein

Each lentiviral construct and the three packaging plasmids (pLP1, pLP2, and pLP/VSVG) (Invitrogen, Carlsbad, CA) were co-transfected into the 293FT cells by lipofectamine 2000 (Invitrogen, Carlsbad, CA) to produce the three kinds of lentivirus: Lenti6.3-Core, Lenti6.3-NS4B and Lenti6.3-mkate2. The titer of the lentivirus was determined by serial dilution and counting the number of mkate2-positive cells in each dilution. The Huh7 and LO2 cells were infected by the lentivirus obtained at a multiplicity of infection (MOI) of 50, respectively, and the infection efficiency was assessed under fluorescent microscope. The Huh7 stable cell lines expressing HCV Core protein, NS4B and mkate2: Huh7-Core, Huh7-NS4B and Huh7-mkate2 were selected using 3 μg/ml blasticidin; the LO2 stable cell lines expressing HCV Core protein, NS4B and mkate2: LO2-Core, LO2-NS4B and LO2-mkate2 were screened using 2 μg/ml blasticidin. The expressions of HCV Core and NS4B mRNAs and proteins in these lentiviral-stable cell lines were detected by real-time qPCR and western blotting.

### Dual-luciferase reporter assay

To detect β-catenin/Tcf transcriptional activity, we utilized the luciferase reporter plasmids pTOPFLASH with wild-type TCF binding sites (Upstate Biotechnology, Lake Placid, NY, USA) and pFOPFLASH with mutant TCF binding sites (Upstate Biotechnology, Lake Placid, NY), each used for the non-specific activation of the reporter. The pRL-TK-containing *Renilla* luciferase vector (Promega, Madison, WI, USA) was used as an internal control for transfection efficiency. The LO2-Core, LO2-NS4B, and LO2-mkate2 were seeded into 6-well plates at a density of 5.0 × 10^5^ cells/well, with each cell type inoculated into three wells. When the cells had adhered to the wells, the LO2 cells were treated with complete medium with or without Wnt3a (R&D Systems, Inc., Minneapolis, USA) at concentrations of 100 and 200 ng/mL for 24 h, then co-transfected with 1.8 μg of pTOPFLASH or pFOPFLASH and 0.2 μg of pRL-TK using Lipofectamine 2000 (Invitrogen, Carlsbad, CA). The Huh7-Core, Huh7-NS4B, and Huh7-mkate2 were seeded into 6-well plates at a density of 5.0 × 10^5^ cells/well, grown to 80–90% confluence, then co-transfected with 1.8 μg of pTOPFLASH or pFOPFLASH and 0.2 μg of pRL-TK. Forty-eight hours after transfection, these cells were lysed, and luciferase activity was detected using a Dual-Luciferase Reporter Assay System (Promega). The experiment was repeated three times. β-catenin/Tcf transcriptional activity was expressed as the relative luciferase activity of pTOP/pFOP normalized to *Renilla* luciferase activity.

### Indirect immunofluorescence

Huh7-Core, Huh7-NS4B, Huh7-mkate2, LO2-Core, LO2-NS4B, and LO2-mkate2 were seeded into 35-mm confocal culture dishes at a density of 2000 cells per well, with each LO2 cell type inoculated into three dishes. When the LO2 cells had adhered to the wells, 100 ng/mL Wnt3a, 200 ng/mL Wnt3a, and fresh complete medium were added to each of the LO2 plates, which were incubated for 24 h; when the Huh7 cells reached 70–80% confluence, the medium was discarded and the cells were washed with phosphate-buffered saline (PBS; Shanghai Sangon Biological Technology and Services Co., Ltd., Shanghai, China), followed by 15 min of fixation in 4% paraformaldehyde (Sinopharm Chemical Reagent Co., Ltd., Shanghai, China), a rinse, 10 min of incubation with 0.3% Triton X-100 (Amresco, Solon, OH, USA) at room temperature, a rinse, 60 min of blocking in 2% bovine serum albumin (BSA; Genview, Florida, USA) at room temperature, and overnight incubation in diluted β-catenin antibody (cat. no. ab16051, 2% BSA, 1:100; Abcam, Cambridge, UK) at 4 °C. The rinsed cells were then incubated with diluted CF 488A–labeled secondary antibody (cat. no. SAB4600045, 2% BSA, 1:50; Sigma-Aldrich, St. Louis, MO, USA) at 37 °C in darkness for 1 h, followed by 5 min of incubation in 4′,6-diamidino-2-phenylindole (DAPI) (1:1000; Beyotime Institute of Biotechnology, Jiangsu, China). A fluorescence microscope (Olympus IX71, Olympus Corp., Tokyo, Japan) was then used to examine the cells and obtain images. ImageJ software (National Institutes of Health, USA) was used to quantify the fluorescence. A region of interest (ROI) was randomly selected in the nucleus and cytoplasm, and the images were transformed to 8-bit color. The ratio of the density of staining in the nucleus versus the cytoplasm was determined by measuring the integrated density of the ROIs in the nucleus and cytoplasm. Cells were determined to be positive for nuclear β-catenin based on the density ratio: a value equal to or less than 1 was considered negative, whereas a value more by greater was considered positive. At least 200 cells were counted in three different microscopic fields for each cell type.

### Real-time quantitative PCR (qPCR)

TRIzol reagent (Invitrogen) was used to extract total RNA from the above cells. A SuperScript III First-Strand Synthesis System (Invitrogen) was then used to reverse transcribe 2 μg of the total RNA into complementary DNA (cDNA). The synthesized cDNA was used as a template for detection of core, NS4B, wnt1, β-catenin, cyclin D1, and c-myc mRNA expression. The primer sequences (Invitrogen) used were as follows. Core: 5′-CACAAATCCTAAACCCCAAAGA-3′ (forward) and 5′-TAGTTCACGCCGTCCTCCA-3′ (reverse); NS4B: 5′-TGGCATTCACAGCCTCTATCAC-3′ (forward) and 5′-GCAGGGAGTAGATTGAGCAGGT-3′ (reverse); wnt1: 5′-GATGGTGGGGTATTGTGAACG-3′ (forward) and 5′-GGAGGTGATAGCGAAGATAAACG-3′ (reverse); β-catenin: 5′-ACAGGAAGGGATGGAAGGTCTC-3′ (forward) and 5′-CTCACGCAAAGGTGCATGATTT-3′ (reverse); cyclin D1: 5′-GGCCACTTGCATGTTCGTG-3′ (forward) and 5′-TGCGGATGATCTGTTTGTTCTC-3′ (reverse); c-myc: 5′-GAGTTTCATCTGCGACCCG-3′ (forward) and 5′-CCAGGAGCCTGCCTCTTTT-3′ (reverse); and GAPDH: 5′-GAAGGTCGGAGTCAACGGATT-3′ (forward) and 5′-CGCTCCTGGAAGATGGTGAT-3′ (reverse). The PCR mixture, 20 μL in total, comprised 2 μL of 10× PCR buffer, 1 μL of MgCl_2_ (50 mM), 0.5 μL of dNTPs (10 mM), 0.5 μL each of forward and reverse primer (10 μM), 0.3 μL of SYBR green (20×), 0.2 μL of Taq DNA polymerase (5 U/μL), 1 μL of cDNA, and 14 μL of nuclease-free water. The PCR conditions were: 40 cycles of 95 °C for 2 min; and 95 °C for 10 s, 60 °C for 30 s, and 70 °C for 45 s. All qPCRs were performed in triplicate on a 7500 Fast Real-Time PCR System (Applied Biosystems, Foster City, CA, USA). The relative expression level of each gene was calculated using the 2^−ΔΔCt^ method, with ΔΔCt = △Ct of the test sample − △Ct of the control sample and ΔCt = mean Ct of the test gene − mean Ct of GAPDH.

### Western blotting

The above cells were lysed using a radioimmunoprecipitation assay buffer (Beyotime Institute of Biotechnology, Jiangsu, China) to obtain total cellular proteins after centrifugation. The extraction of nuclear and cytoplasmic protein was performed using NE-PER nuclear and cytoplasmic extraction reagents (Thermo Scientific Pierce, Rockford, IL, USA) according to the manufacturer’s protocol. A Bradford Protein Assay Kit (Beyotime Institute of Biotechnology, Jiangsu, China) was then used to detect protein concentrations. Sixty micrograms of protein sample was then loaded onto a gel for 12% sodium dodecyl sulfate-polyacrylamide gel electrophoresis (SDS-PAGE) to separate the proteins. The proteins were then transferred onto semi-dry polyvinylidene difluoride (PVDF) membranes (Bio-Rad Laboratories, Inc., Hercules, CA, USA) that had been blocked for 2 h using 5% skim milk (Beyotime Institute of Biotechnology) in Tris-buffered saline with 0.1% Tween-20 (Sigma-Aldrich, St. Louis, MO, USA). The proteins were then cultured overnight in the following antibodies at 4 °C: mouse anti-HCV core 1b antibody (cat. no. ab2740, 1:1000 dilution; Abcam, Cambridge, UK), mouse anti-HCV NS4B antibody (cat. no. ab24283, 1:1000 dilution; Abcam), rabbit anti-beta catenin antibody (cat. no. ab16051, 1:1000 dilution; Abcam), goat anti-wnt1 antibody (cat. no. sc-6266, 1:1000 dilution; Santa Cruz Biotechnology, Inc., Dallas, TX, USA), β-actin antibody (cat. no. sc-47,778, 1:1000 dilution; Santa Cruz Biotechnology, Inc.), GAPDH antibody (cat. no. AF0006, 1:1000 dilution; Beyotime Institute of Biotechnology, Jiangsu, China), rabbit anti-c-myc antibody (cat. no. bs-4963R, 1:100 dilution; Bioss Biotechnology, Inc., Beijing, China), rabbit anti-cyclin D1 antibody (cat. no. bs-0623R, 1:100 dilution; Bioss Biotechnology, Inc.), rabbit anti-beta tubulin antibody (cat. no. bs-0210R, 1:100 dilution; Bioss Biotechnology, Inc.), and rabbit anti-nucleophosmin antibody (B23) (cat. no. bs-4757R, 1:100 dilution; Bioss Biotechnology, Inc.). After washing, the membrane was incubated with the following secondary antibodies: horseradish peroxidase (HRP)-conjugated goat anti-rabbit immunoglobulin G (IgG) (cat. no. A0208, 1:5000 dilution; Beyotime Institute of Biotechnology), HRP-conjugated goat anti-mouse IgG (cat. no. A0216, 1:5000 dilution; Beyotime Institute of Biotechnology), and HRP-conjugated donkey anti-goat IgG (cat. no. A0181, 1:5000 dilution; Beyotime Institute of Biotechnology) at room temperature for 2 h. A BeyoECL Plus Enhanced Chemiluminescent Kit (Beyotime Institute of Biotechnology) was then used to test the proteins, and Alpha Imager (IS-2200) software (Alpha Innotech Corporation, San Leandro, CA, USA) was used to semi-quantitatively analyze the band densities. B23, β-tubulin, and β-actin (or GAPDH) were used as loading controls for the nuclear proteins, cytoplasmic proteins, and total cellular proteins, respectively. The relative protein expression level was a density ratio of each protein band relative to its loading control band.

### 3-(4,5-dimethylthiazol-2-yl)-2,5-diphenyltetrazolium bromide (MTT) assay

The cells described above were seeded into 96-well plates at a density of 2000 cells per well, and continuously paved the plates for five days. MTT assays were then performed at five time-points (24, 48, 72, 96, and 120 h). MTT (10 μL, 5 mg/mL; Beyotime Institute of Biotechnology) was added to each well, and the plates were incubated for 4 h. Formazan lysate (100 μL) was then added to each well followed by incubation for another 4 h at 37 °C. The absorbance values were then measured at 570 nm.

### Plate clone assay (PCA)

The cells described above were seeded into 6-well plates at a density of 5.0 × 10^5^ cells per well. When the cells had adhered to the wells, the LO2 cells were divided into two groups: an inducer-free group and a 200 ng/mL Wnt3a group, and the cells in each group were cultured for 24 h; when the Huh7 cells reached 80% confluence, 0.25% trypsin (Gibco) was used to digest and collect the cells, which were then added to 10% FBS-containing RPMI 1640 or DMEM to produce a cell suspension with a final concentration of 1.0 × 10^4^ cells/mL. One hundred and fifty microliters of the above cell suspension was then inoculated into new 6-well plates, 2.5 mL of 10% FBS-containing RPMI 1640 or DMEM was added, and the cells were cultured for 2 weeks at 37 °C and 5% CO_2_. After fixation with 500 μL of methanol (Sinopharm Chemical Reagent Co., Ltd., Beijing, China) for 15 min, 800 μL of Giemsa staining liquid (Sinopharm Chemical Reagent Co., Ltd., Beijing, China) was added to the cell suspension, and the cells were cultured for 30 min. The number of cell clones was determined, and the cloning efficiency was calculated using the following equation: (%) = number of clones/inoculated cells × 100%. The experiment was repeated three times.

### Cell cycle analysis

Huh7 and LO2 cells in the logarithmic phase were cultivated for 24 h in DMEM or RPMI 1640 nutrient solution without serum. The cells were then collected by 0.25% trypsinization and centrifugation at 1000 rpm. The collected cells were suspended in nutrient solution with 10% fetal calf serum, and inoculated into 6-well plates at a concentration of 5 × 10^5^ cells. When the cells had adhered to the wells, the LO2 cells was divided into two groups: an inducer-free group and a 200 ng/mL Wnt3a group, and the cells in each group were cultured for 24 h; when the Huh7 cells had reached 80–90% confluence, the cells in each group were digested with 0.25% trypsin (Gibco), collected, centrifuged at 2000 rpm for 5 min, rinsed in PBS, and fixed overnight in pre-cooled 70% ethanol (Sinopharm Chemical Reagent Co., Ltd., Shanghai, China) at 4 °C. The cells were then collected, and 5 μL of RNase A (Sigma-Aldrich) and 10 μL of propidium iodide (PI) (Becton Dickinson, Franklin Lakes, NJ, USA) were added and mixed. This was followed by incubation in darkness for 30 min. Subsequently, PI signal was detected at 617 nm wavelength using BD FACSCalibur system (Becton Dickinson, Franklin Lakes, NJ, USA) within an hour, and the cell cycle phase deduced from the fluorescence intensity using CellQuest software (Becton Dickinson).

### Statistical analysis

SPSS 16.0 software (SPSS Inc., Chicago, IL, USA) was used for the statistical analysis. All data are expressed as the mean ± standard deviation (SD). One-way analysis of variance was used for comparisons of multiple groups, and Student–Newman–Keuls and least significant difference tests were used for pairwise comparisons of multiple groups, with *P* < 0.05 considered a statistically significant difference.

## Results

### Expression of HCV core protein and NS4B in Huh7 and LO2 cells

**Fig. 1:**
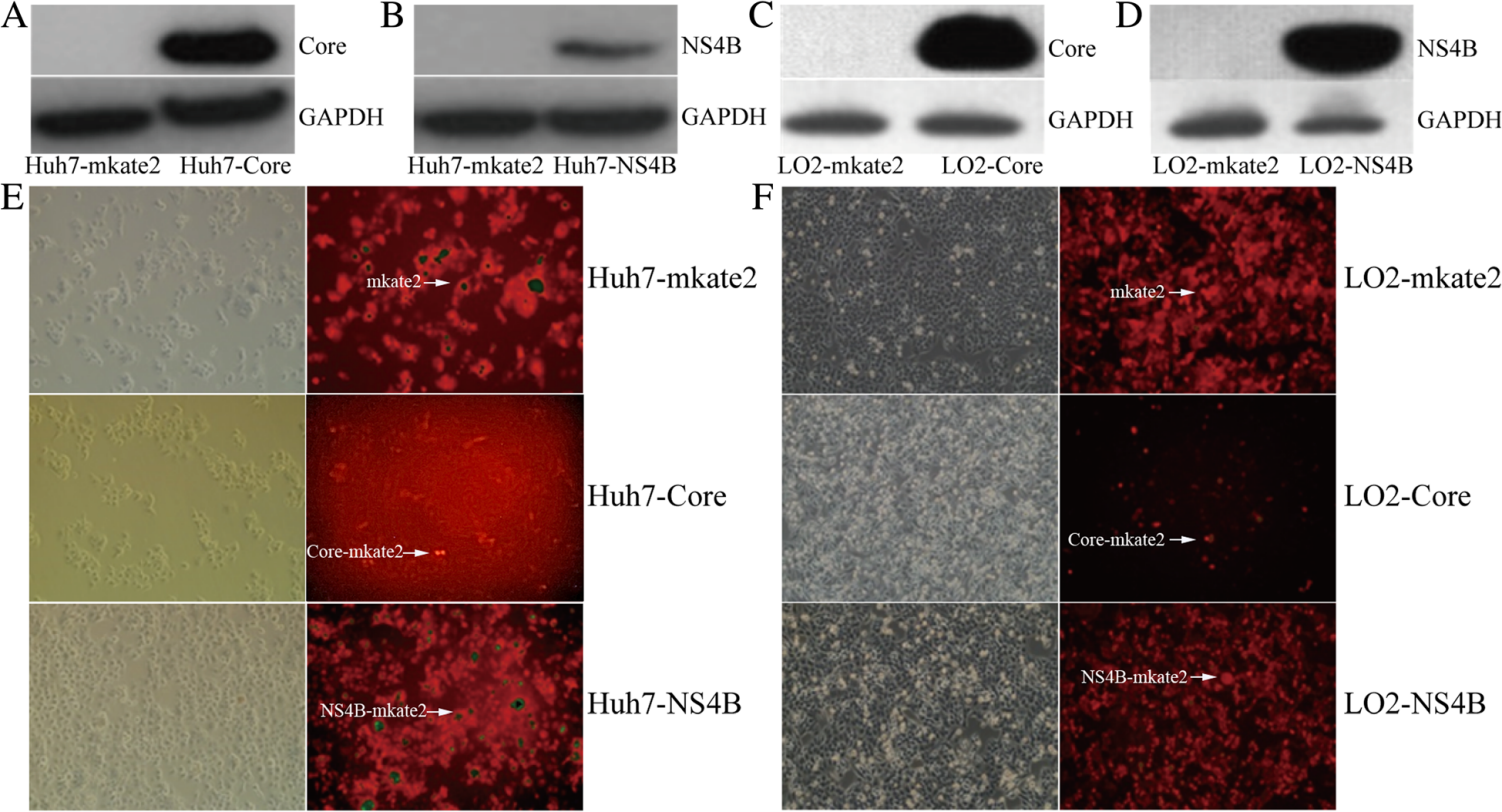
Expression of HCV core protein and NS4B in Huh7 and LO2 cells. **a** Over-expression of HCV core protein in Huh7-Core cells; **b** Over-expression of HCVNS4B in Huh7-NS4B cells; **c** Over-expression of HCV core protein in LO2-Core cells; **d** Over-expression of HCVNS4B in LO2-NS4B cells; **e** Visible light and fluorescent light in Huh7 cells (×100); **f** Visible light and fluorescent light in LO2 cells (×100)

The pLenti6.3-Core, pLenti6.3-NS4B and pLenti6.3-mkate2 expressing HCV Core, NS4B and mkate2 were successfully constructed (Additional file 2: Figure S1). Real-time qPCR was used to investigate the expression of HCV core and NS4B mRNAs, and western blotting was used to investigate the expression of HCV core and NS4B proteins in the stable cell lines. The mRNA expression levels in the Huh7-Core and Huh7-NS4B cells were 183.71 ± 10.80 and 85.16 ± 5.60 greater than in the Huh7-mkate2 cells, respectively. The HCV core and NS4B mRNA expression levels in the LO2-Core and LO2-NS4B cells were 94.76 ± 16.93 and 2.12 × 10^5^ ± 1.68 × 10^4^ greater than in the LO2-mkate2 cells, respectively. Western blotting revealed the presence of the core protein (21 kDa) in the Huh7-Core cells and NS4B (27 kDa) in the Huh7-NS4B cells, whereas those proteins were not detected in the Huh7-mkate2 cells (Fig. 1a-b). Similar results were obtained for the LO2-Core and LO2-NS4B cells (Fig. 1c-d). The Huh7 and LO2 cells were visualized with visible light and fluorescence images (×100, Fig. 1e–f).

### HCV core protein and NS4B enhance β-catenin/Tcf transcriptional activities in Huh7 cells or LO2 cells following Wnt3a induction

**Fig. 2:**
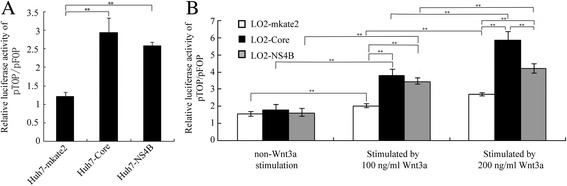
HCV core protein and NS4B enhance the β-catenin/Tcf-dependent transcriptional activity in Huh7 cells or LO2 cells under Wnt3a induction. **a** Relative luciferase activity of pTOP/pFOP in Huh7 cells. HCV core protein and NS4B enhance the β-catenin/Tcf-dependent transcriptional activity in Huh7 stable cell line. Compared with Huh7-mkate2, ^**^ *P* < 0.01. **b** Relative luciferase activity of pTOP/pFOP in LO2 cells non-Wnt3a stimulation, stimulated by 100 ng/ml Wnt3a, stimulated by 200 ng/ml Wnt3a. HCV core protein and NS4B stabilize the β-catenin/Tcf-dependent transcriptional activity in LO2 stable cell line under Wnt3a induction. ^**^ *P* < 0.01

To investigate the effects of HCV core protein and NS4B on β-catenin/Tcf transcriptional activity, we performed dual reporter assays using the pTOPFLASH or pFOPFLASH firefly luciferase reporter and the pRL-TK *Renilla* luciferase vector. The statistical analysis showed that the relative luciferase activity of pTOP/pFOP in the Huh7-Core and Huh7-NS4B cells was higher than in the Huh7-mkate2 cells (2.93 ± 0.40, 2.58 ± 0.09, and 1.21 ± 0.12, *F* = 40.87, *P* < 0.01). When no Wnt3a was used to stimulate the cells, there was no statistically significant difference in the pTOP/pFOP relative luciferase activity between the LO2-Core, LO2-NS4B, and LO2-mkate2 cells (1.78 ± 0.35, 1.61 ± 0.18, and 1.57 ± 0.14, *F* = 0.65, *P* > 0.05). After 100 ng/mL Wnt3a and 200 ng/mL Wnt3a were used to stimulate the LO2 cells, the pTOP/pFOP relative luciferase activity in the LO2-Core and LO2-NS4B cells was significantly higher than in the LO2-mkate2 cells (stimulated with 100 ng/mL Wnt3a: 3.82 ± 0.37, 3.41 ± 0.09, and 2.03 ± 0.12, *F* = 48.69, *P* < 0.01; stimulated with 200 ng/mL Wnt3a: 5.87 ± 0.51, 4.22 ± 0.28, and 2.71 ± 0.11, F = 64.25, *P* < 0.01). Activity was higher in the LO2-Core cells than in the LO2-NS4B cells (*P* < 0.01), and activity in the LO2-NS4B cells was higher than in the LO2-mkate2 cells (*P* < 0.01, Fig. 2).

### HCV core protein and NS4B increase the nuclear translocation of β-catenin in Huh7 cells or LO2 cells following Wnt3a induction

**Fig. 3:**
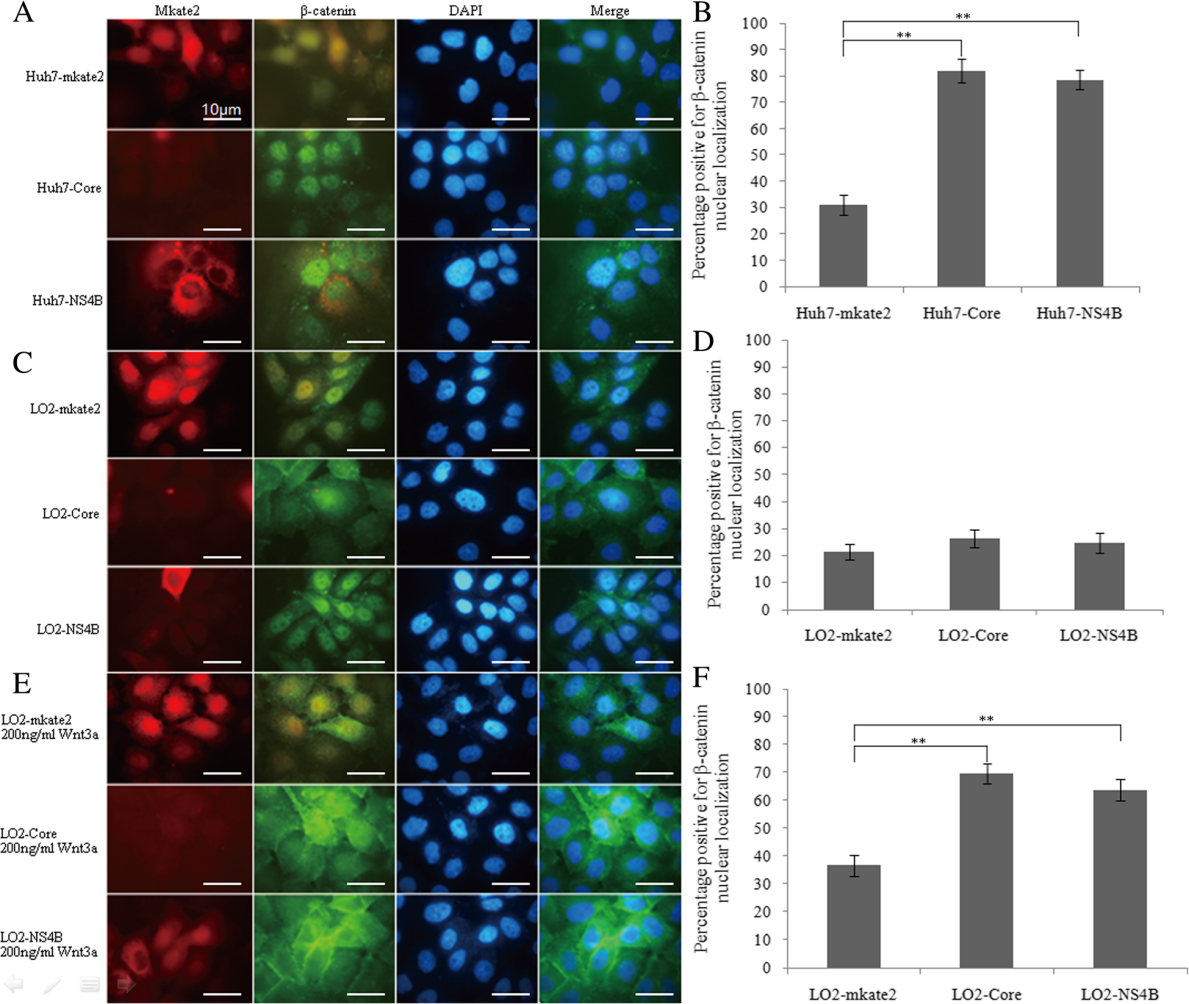
HCV core protein and NS4B promote, directly or under Wnt3a induction, the nuclear translocation of β-catenin in Huh7 cells and LO2 cells. **a** Subcellular localization of β-catenin in Huh7 cells (magnification, ×1000). β-catenin protein was detected by using an anti-β-catenin antibody which was visualized by an anti-rabbit secondary antibody conjugated with CF 488A dye (*green*). Nuclei were counterstained with DAPI (*blue*). **b** The histogram indicating the percentage of positive cells for β-catenin nuclear localization in Huh7 cells. HCV core protein and NS4B promote the nuclear translocation of β-catenin in Huh7 cells. Compared with Huh7-mkate2, **P < 0.01. The percentage of positive cells for β-catenin nuclear staining was the ratio of the nuclear β-catenin positive cells to total number of cells. At least 200 cells were counted from three different microscopic fields for each cell strain. **c** Subcellular localization of β-catenin in LO2 cells (magnification, ×1000). **d** The histogram indicating the percentage of positive cells for β-catenin nuclear localization in LO2 cells with non-Wnt3a stimulation. HCV core protein and NS4B do not increase the nuclear translocation of β-catenin in LO2 cells. Compared with LO2-mkate2, *P* > 0.05. **e** Subcellular localization of β-catenin in LO2 cells under 24 h 200 ng/ml Wnt3a stimulation (magnification, ×1000). **f** The histogram indicating the percentage of positive cells for β-catenin nuclear localization in LO2 cells under 200 ng/mL Wnt3a stimulation. HCV core protein and NS4B increase the nuclear translocation of β-catenin in LO2 cells under Wnt3a stimulation. Compared with LO2-mkate2, **P < 0.01

Indirect immunofluorescence staining was performed to detect the nuclear translocation of β-catenin protein, and the results showed that the percentage of cells positive for the nuclear localization of β-catenin protein in the Huh7-Core and Huh7-NS4B cells was significantly higher than in the Huh7-mkate2 cells (82.00 ± 4.36, 78.67 ± 3.51, and 31.00 ± 3.61, *F* = 165.26, *P* < 0.01) (Fig. 3a-b). When Wnt3a was not used to stimulate the LO2 cells, there was no significant difference between the percentage of cells positive for β-catenin nuclear localization in the LO2-Core and LO2-NS4B cells and the LO2-mkate2 cells (26.33 ± 3.21, 24.67 ± 3.79, and 21.17 ± 2.93, *F* = 1.88, *P* > 0.05) (Fig. 3c-d). When stimulated with two different concentrations of Wnt3a (100 ng/mL and 200 ng/mL) for 24 h, there was a higher percentage of cells positive for the nuclear localization of β-catenin protein in the LO2-Core and LO2-NS4B stable cell lines compared with the LO2-mkate2 cells (stimulated with 200 ng/mL Wnt3a: 69.67 ± 3.51, 63.83 ± 3.75, and 36.83 ± 3.88, F = 66.54, *P* < 0.01) (Fig. 3e-f).

### HCV core protein and NS4B upregulate the expression of β-catenin and its target genes in Huh7 cells but not in LO2 cells

**Fig. 4:**
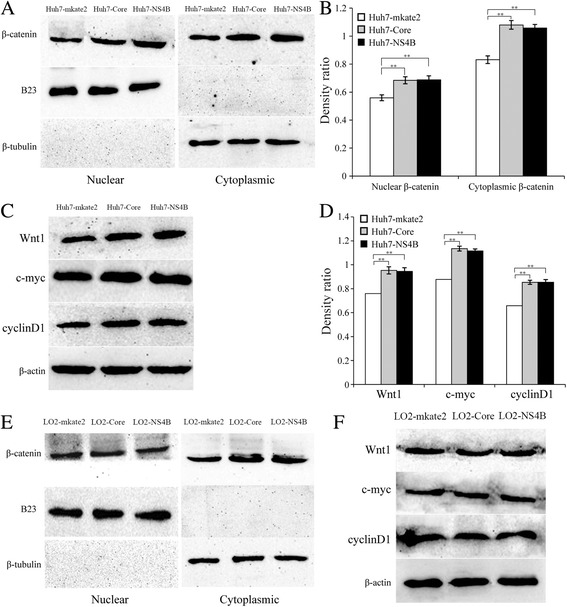
HCV core protein and NS4B up-regulate protein expressions of β-catenin and its target genes in Huh7 cells but not in LO2 cells. **a** Western blotting analysis of nuclear β-catenin and cytoplasmic β-catenin protein expressions in Huh7 cells. B23 and β-tubulin were used as loading controls for the nuclear protein and cytoplasmic protein. **b** The relative protein expression level was a density ratio of each protein band relative to its loading control band. The density ratios of nuclear β-catenin band to B23 band and cytoplasmic β-catenin band to β-tubulin band in Huh7 cells. Compared with Huh7-mkate2, **P < 0.01. **c** Western blotting analysis of protein expressions of Wnt1, c-myc, and cyclinD1 in Huh7 cells. β-actin was used as a loading control for the total cellular protein. **d** The density ratios of Wnt1, c-myc, and cyclinD1 protein bands to β-actin in Huh7 cells. Compared with Huh7-mkate2,**P < 0.01. **e** Western blotting analysis of protein expressions of nuclear β-catenin and cytoplasmic β-catenin in LO2 cells. B23 and β-tubulin were used as loading controls for the nuclear protein and cytoplasmic protein. Analysis of the density ratios of nuclear β-catenin band to B23 and cytoplasmic β-catenin band to β-tubulin in LO2 cells show that there was no significant difference in the relative protein expression levels of nuclear β-catenin and cytoplasmic β-catenin among LO2-Core, LO2-NS4B, and LO2-mkate2 (*P* > 0.05). **f** Western blotting analysis of protein expressions of Wnt1, c-myc, and cyclinD1 in LO2 cells. β-actin was used as a loading control for the total cellular protein. Analysis of the density ratios of Wnt1, c-myc, and cyclinD1 protein bands to β-actin in LO2 cells show that there was no significant difference in the relative protein expression levels of Wnt1, c-myc, and cyclinD1 among LO2-Core, LO2-NS4B, and LO2-mkate2 (*P* > 0.05)

The relative mRNA and protein expression levels of β-catenin, Wnt1, c-myc, and cyclin D1 in each Huh7 cell line were determined by real-time qPCR and western blotting. The results showed that the relative mRNA expression levels of Wnt1 and cyclin D1 in the Huh7-Core and Huh7-NS4B cells were slightly higher than those in the Huh7-mkate2 cells (Wnt1 mRNA: 2.19 ± 0.48, 1.81 ± 0.45, *F* = 7.57, *P* < 0.05; cyclin D1 mRNA: 2.20 ± 0.46, 1.67 ± 0.34, *F* = 9.87, *P* < 0.05). The relative mRNA expression levels of β-catenin and c-myc in the Huh7-Core and Huh7-NS4B cells were not significantly different from those in the Huh7-mkate2 cells (both *P* > 0.05). The relative protein expression level was a density ratio of each protein band relative to its loading control band. The relative protein expression levels of β-catenin in the nucleus and cytoplasm and Wnt1, c-myc, and cyclin D1 in the Huh7-Core and Huh7-NS4B cells were significantly higher than those in the Huh7-mkate2 cells (the density ratios of nuclear β-catenin band to B23 band: 0.68 ± 0.03, 0.69 ± 0.03, and 0.56 ± 0.02, *F* = 26.59, *P* < 0.01; the density ratios of cytoplasmic β-catenin band to β-tubulin band: 1.08 ± 0.03, 1.06 ± 0.03, and 0.83 ± 0.03, *F* = 73.65, *P* < 0.01; the density ratios of Wnt1 band to β-actin band: 0.95 ± 0.03, 0.94 ± 0.03, and 0.76 ± 0.03, *F* = 39.55, *P* < 0.01; the density ratios of c-myc band to β-actin band: 1.14 ± 0.02, 1.12 ± 0.02, and 0.88 ± 0.03, *F* = 144.85, *P* < 0.01; the density ratios of cyclin D1 band to β-actin band: 0.85 ± 0.02, 0.85 ± 0.02, and 0.66 ± 0.02, *F* = 91.88, *P* < 0.01) (Fig. 4a–d). These results suggest that HCV core protein and NS4B promote the expression of β-catenin, Wnt1, c-myc, and cyclin D1 proteins in Huh7 cells.

However, there was no significant difference in the relative mRNA and protein expression levels of β-catenin, Wnt1, c-myc, and cyclin D1 between the LO2-Core, LO2-NS4B, and LO2-mkate2 cells (*P* > 0.05) (Fig. 4e–f). These results suggest that HCV core protein and NS4B do not directly upregulate the mRNA and protein expression levels of β-catenin, Wnt1, c-myc, and cyclin D1 in LO2 cells.

### HCV core protein and NS4B promote the proliferation of Huh7 cells or LO2 cells following Wnt3a induction

**Fig. 5:**
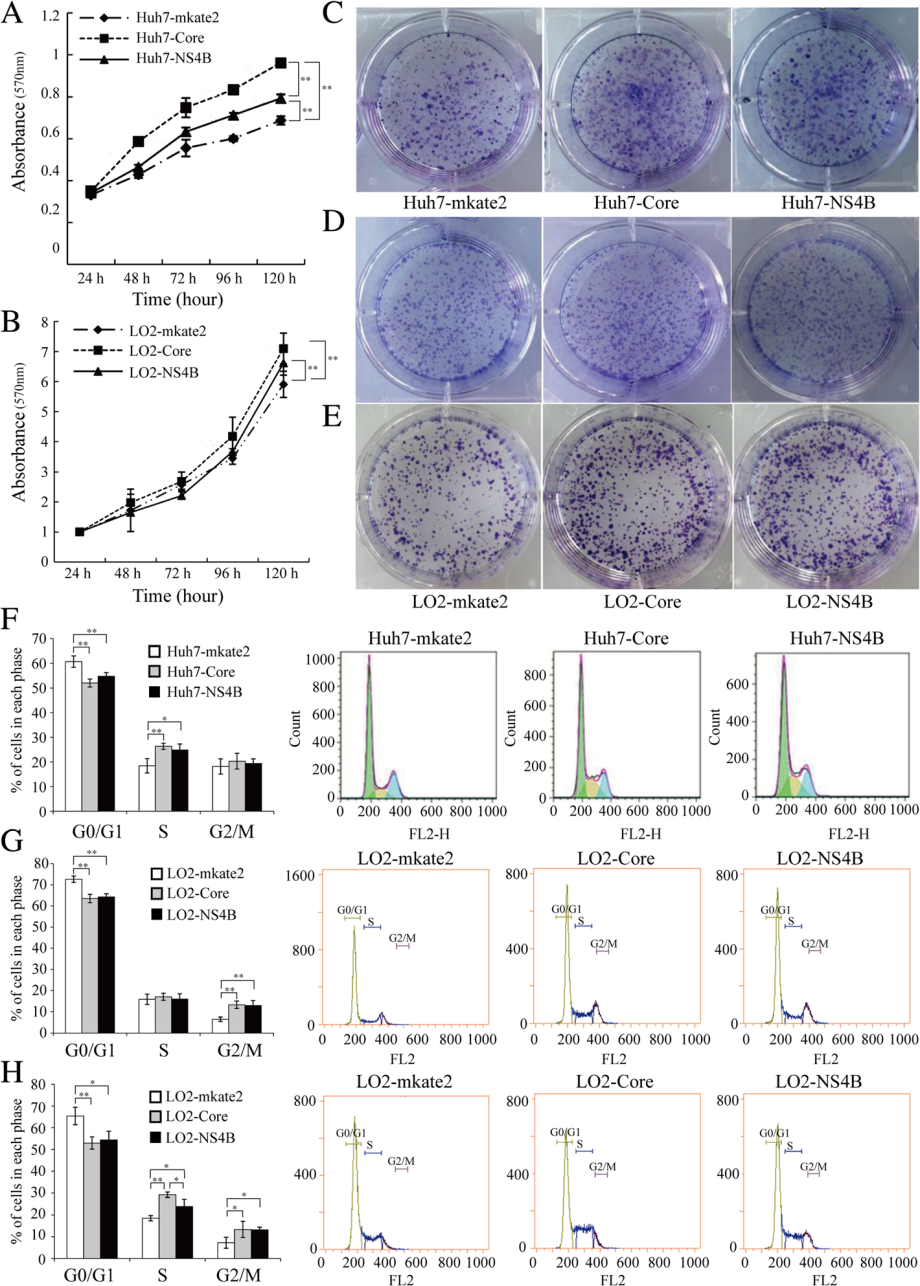
HCV core protein and NS4B promote the proliferation of Huh7 cells or the proliferation of LO2 cells under Wnt3a induction. **a** Cell growth curves of Huh7 strains. Compared with Huh7-mkate2 or Huh7-NS4B, ^**^ *P* < 0.01. **b** Cell growth curves of LO2 strains. Compared with LO2-mkate2, **P < 0.01. **c** PCA of Huh7 strains. **d** PCA of LO2 strains. **e** PCA of LO2 strains after stimulated using 200 ng/ml Wnt3a. **f** Cell cycle results of Huh7 strains. Compared with Huh7-mkate2, **P < 0.01, *P < 0.05. **g** Cell cycle results of LO2 strains. Compared with LO2-mkate2, **P < 0.01. **h** Cell cycle results of LO2 strains after 24-h stimulation using 200 ng/ml Wnt3a. Compared with LO2-mkate2 or LO2-NS4B, **P < 0.01, *P < 0.05

The MTT assay results showed that the Huh7-Core cells proliferated faster than the Huh7-NS4B cells (*P* < 0.01), and that the proliferation of the Huh7-NS4B cells was faster than that of the Huh7-mkate2 cells (*P* < 0.01). We found similar results for the LO2 cells (Fig. 5a-b).

PCA revealed the cloning efficiencies of the Huh7-mkate2, Huh7-Core, and Huh7-NS4B cells to be 26.40% ± 3.17%, 52.27% ± 3.25%, and 42.93% ± 2.38%, respectively (*F* = 58.71, *P* < 0.01) (Fig. 5c). The cloning efficiencies of the LO2-mkate2, LO2-Core, and LO2-NS4B cells were 31.30% ± 2.25%, 36.00% ± 2.78%, and 35.83% ± 2.10%, respectively (*F* = 3.71, *P* > 0.05) (Fig. 5d). When 200 ng/mL Wnt3a was used to stimulate the LO2 cells, the cloning efficiencies in the LO2-mkate2, LO2-Core, and LO2-NS4B cells were 32.37% ± 3.07%, 47.50% ± 4.27%, and 45.60% ± 4.14%, respectively (*F* = 13.66, *P* < 0.01) (Fig. 5e).

Flow cytometry was performed to determine the cell cycle status of the Huh7-Core, Huh7-NS4B, and Huh7-mkate2 cells, and the results (Fig. 5f) showed that the ratio of cells in the G0/G1 phase in the Huh7-Core and Huh7-NS4B cells was reduced relative to that in the Huh7-mkate2 cells (52.01 ± 1.61, 54.77 ± 1.42, and 60.69 ± 2.33, *F* = 17.63, *P* < 0.01), but the proportion of cells in the S phase in the Huh7-Core and Huh7-NS4B cells increased relative to that in the Huh7-mkate2 cells (26.35 ± 1.28, 24.87 ± 2.41, and 18.46 ± 2.90, *F* = 9.98, *P* < 0.05). The proportion of cells in the S phase in the LO2-Core and LO2-NS4B cells was not significantly different from that in the LO2-mkate2 cells (*P* > 0.05) (Fig. 5g).

The results of 24-h stimulation with 200 ng/mL Wnt3a in the LO2 cell lines showed that the proportion of cells in the G0/G1 phase in the LO2-Core and LO2-NS4B cells was reduced relative to that in the LO2-mkate2 cells (52.95 ± 2.81, 54.39 ± 3.97, and 65.39 ± 4.01, *F* = 10.50, *P* < 0.05), but the proportion of cells in the S and G2/M phases increased significantly (S phase: 29.22 ± 1.20, 23.90 ± 3.29, and 18.52 ± 1.19, *F* = 18.86, *P* < 0.01; G2/M phase: 13.37 ± 3.71, 13.15 ± 1.29, and 7.25 ± 2.54, *F* = 4.96, *P* < 0.05). The proportion of cells in the S phase was higher in the LO2-Core cells than in the LO2-NS4B cells (29.22 ± 1.20 versus 23.90 ± 3.29, *P* < 0.05) (Fig. 5h).

## Discussion

The excessive activation of the Wnt/β-catenin signaling pathway is associated with the occurrence of HCC. In HCC tissues, various molecules in the Wnt/β-catenin signaling pathway, such as the scaffold protein axin, β-catenin protein, and secreted frizzled-related protein 1 (SFRP1), have shown abnormal expression [18, 19]. Studies have shown that the HCV core protein and NS4B have the potential to transform cells [11, 20], but whether these two proteins affect the Wnt/β-catenin signaling pathway and induce HCC remains unclear.

Analysis of the different gene expression profiles between LO2-Core and LO2-mkate2, as well as LO2-NS4B and LO2-mkate2, were conducted with cDNA microarrays; and the significant pathways of the differential genes involved were selected by the Fisher’s exact test and χ^2^ test according to kyoto encyclopedia of genes and genomes (KEGG) database. The results showed that HCV Core protein and NS4B did not increase the activity of Wnt/beta-catenin signaling pathway in LO2 cells, which was inconsistent with previous investigations [14, 15, 21]. Then we conducted Wnt3a induced LO2 cells group and non-induced LO2 cells group. Since Liu et al. [15] reported that in Huh7 cells, HCV core protein enhanced Wnt3a-induced β-catenin/Tcf-dependent transcriptional activity in a dose-dependent manner, but core alone exhibited limited activation of the pTOPFLASH luciferase reporter in Huh7 cells, so the stimulation of HCV Core and NS4B in Huh7 by Wnt/β-catenin signaling pathway still needs further study to confirm.

In the current study, we found that HCV core protein and NS4B in Huh7 cells enhance the transcriptional activity of β-catenin/Tcf, promote the nuclear translocation of β-catenin, upregulate wnt1, β-catenin, c-myc, and cyclin D1 protein levels, and promote the proliferation of Huh7 cells, which showed that HCV core protein and NS4B would activate Wnt/β-catenin signaling pathway in Huh7 cells. Furthermore, the results of this study showed that HCV core protein and NS4B did not significantly increase the mRNA levels of β-catenin and c-myc in the Huh7 cells. This suggests that the upregulation of β-catenin and c-myc expression in Huh7 cells by these two proteins occurs at the post-transcriptional level. This corroborates the results of a study on the activation of the β-catenin signaling cascade by HCVNS5A protein [22]. Activation of the Wnt/β-catenin signaling pathway by NS4B has seldom been reported. Moreover, comparative studies on the impact of HCV core protein and NS4B on the Wnt/β-catenin signaling pathway are rare. In another study on the HCV-activated Wnt/β-catenin signaling pathway, Quan et al. [23] reported that HCV core protein can induce hypermethylation of the Wnt antagonist SFRP1 promoter, thereby downregulating SFRP1 in Huh7 and HepG2 cells, and activating the Wnt/β-catenin signaling pathway. Huang et al. [24] also reported that HCV core protein can downregulate the expression of microRNA-152, thereby upregulating Wnt1 and promoting the proliferation of HepG2 cells. Zhang et al. [25] reported that HCV can upregulate microRNA-155 to activate the Wnt/β-catenin signaling pathway, and promote liver cell proliferation and tumor formation, with adenomatous polyposis coli (APC) as the direct functional target of microRNA-155.

However, the current study showed that HCV core protein and NS4B did not activate the Wnt/β-catenin signaling pathway in LO2 cells, and did not upregulate β-catenin protein levels in the nucleus and cytoplasm of LO2 cells, or the protein levels of upstream and downstream genes (Wnt1, c-myc, and cyclin D1), indicating that these two proteins in LO2 cells alone cannot activate the Wnt/β-catenin signaling pathway. After stimulation with Wnt3a, the three LO2 cell types showed increased transcriptional activity of β-catenin/Tcf, but HCV core protein and NS4B enhanced Wnt3a-induced β-catenin/Tcf-dependent transcriptional activity. The results of an indirect immunofluorescence assay also revealed that HCV core protein and NS4B expression corresponds to a higher percentage of cells that are positive for the nuclear translocation of β-catenin. The role of HCV core protein was more obvious in these cells, with HCV core protein and NS4B in LO2 cells potentiating Wnt/β-catenin signaling activity following Wnt3a induction, and possibly playing a partial role in the pathogenesis of HCV-related HCC. These results also prompted us to speculate that, in addition to viral factors, the occurrence of HCV-related HCC may be precipitated by host genetics and environmental factors [26–28].

In the current study, compared with NS4B, HCV core protein exhibited a stronger ability to enhance β-catenin/Tcf-dependent transcriptional activity, induce the nuclear translocation of β-catenin, and promote cell proliferation, both in Huh7 cells and in Wnt3a-induced LO2 cells. This indicates that the impact of HCV core protein on the Wnt/β-catenin signaling pathway is stronger than that of NS4B.

The Huh7 cells used in this study constitute a hepatoma cell line that can support the replication of HCV [29]; therefore, they have been widely used as an infected cell culture model for liver-related diseases. One study showed that Huh7 cells express a mutant p53 gene that is transcriptionally inactive; thus, they are not suitable for many studies related to HCV-associated carcinogenesis [28]. Because HCC cell lines are characterized by abnormal proliferation and gene expression compared with normal human liver cells, a normal human liver LO2 cell model was established that could stably express HCV core protein and NS4B. The model better reflects the interactions between HCV proteins and host cells, and facilitates an examination of the effects of HCV proteins on cell signaling, liver cell proliferation, and carcinogenesis. However, the LO2 cells used in this study were transformed normal human liver cells, and HCV core protein and NS4B were both overexpressed in the LO2 cells; therefore, this LO2 cell model cannot fully replicate the molecular mechanisms and interactions between viral proteins and host cells in vivo. Elucidation of the roles of HCV core protein and NS4B in Wnt/β-catenin-induced HCC will require further in vivo experiments. Models of natural HCV infection in vivo and in vitro have many advantages, but also present many challenges. A previously established in vitro model does not completely replicate events in HCV infection in vivo, for example. Adult primary liver cells infected with HCV are used as a model in an environment that is similar to the physiological environment of the liver, but these cells are difficult to handle, replication is low, and the cells do not support long-term HCV replication. Therefore, the applicability of the model is limited. The effects of HCV infection on Wnt pathway signaling in vivo and in vitro require further study.

## Conclusions

In conclusion, our results suggest that HCV core protein and NS4B can trigger Wnt/β-catenin signaling in Huh7 cells, thereby promoting their proliferation. In LO2 cells, these two proteins do not activate the Wnt/β-catenin signaling pathway alone. However, they enhance Wnt3a-induced Wnt/β-catenin signaling, and play a partial roles in the pathogenesis of HCV-related HCC.

## Additional files

Additional file 1: Table S1. Primers for PCR. (DOCX 17 kb)

Additional file 2: Figure. S1. Sequencing results of the pLenti6.3-Core, pLenti6.3-NS4B and pLenti6.3-mkate2 (partial). (DOCX 274 kb)

## Abbreviations

APC: Adenomatous polyposis coli
cDNA: Complementary DNA
DAPI: 4′,6-diamidino-2-phenylindole
DMEM: Dulbecco’s modified Eagle’s medium
FBS: Fetal bovine serum
GSK-3β: Glycogen synthase kinase-3β
HCC: Hepatocellular carcinoma
HCV: Hepatitis C virus
HRP: Horseradish peroxidase
IgG: Immunoglobulin G
MOI: Multiplicity of infection
MTT: 3-(4,5-dimethylthiazol-2-yl)-2,5-diphenyltetrazolium bromide
MW: Membranous web
NS4B: Nonstructural protein 4B
PBS: Phosphate-buffered saline
PCA: Plate clone assay
PCR: Polymerase chain reaction
PI: Propidium iodide
PVDF: Polyvinylidene difluoride
real-time qPCR: real-time quantitative polymerase chain reaction
RPMI 1640: Roswell Park Memorial Institute 1640 medium
RT-PCR: Reverse transcription–polymerase chain reaction
SDS-PAGE: Sodium dodecyl sulfate–polyacrylamide gel electrophoresis
SFRP1: Secreted frizzled-related protein 1

## References

[1] MohdHanafiah K, Groeger J, Flaxman AD, Wiersma ST (2013). Global epidemiology of hepatitis C virus infection: new estimates of age-specific antibody to HCV seroprevalence. Hepatology.

[2] Kim MN, Kim BK, Han KH (2013). Hepatocellular carcinoma in patients with chronic hepatitis C virus infection in the Asia-Pacific region. J Gastroenterol.

[3] Mileo AM, Mattarocci S, Matarrese P, Anticoli S, Abbruzzese C, Catone S (2015). Hepatitis C virus core protein modulates pRb2/p130 expression in human hepatocellular carcinoma cell lines through promoter methylation. J ExpClin Cancer Res.

[4] Moriya K, Fujie H, Shintani Y, Yotsuyanagi H, Tsutsumi T, Ishibashi K (1998). The core protein of hepatitis C virus induce hepatocellular carcinoma in transgenic mice. Nat Med.

[5] Paul D, Hoppe S, Saher G, Krijnse-Locker J, Bartenschlager R (2013). Morphological and biochemical characterization of the membranous hepatitis C virus replication compartment. J Virol.

[6] Gouttenoire J, Montserret R, Paul D, Castillo R, Meister S, Bartenschlager R (2014). Aminoterminal amphipathic α-helix AH1 of hepatitis C virus nonstructural protein 4B possesses a dual role in RNA replication and virus production. PLoSPathog.

[7] Egger D, Wölk B, Gosert R, Bianchi L, Blum HE, Moradpour D (2002). Expression of hepatitis C virus proteins induces distinct membrane alterations including a candidate viral replication complex. J Virol.

[8] Wang H, Tai AW (2016). Mechanisms of cellular membrane reorganization to support hepatitis C virus replication. Viruses..

[9] Thompson AA, Zou A, Yan J, Duggal R, Hao W, Molina D (2009). Biochemical characterization of recombinant hepatitis C virus nonstructural protein 4B: evidence for ATP/GTP hydrolysis and adenylate kinase activity. Biochemistry.

[10] Wang AG, Moon HB, Kim JM, Hwang SB, Yu DY, Lee DS (2006). Expression of hepatitis C virus nonstructural 4B in transgenic mice. ExpMol Med.

[11] Einav S, Sklan EH, Moon HM, Gehrig E, Liu P, Hao Y (2008). The nucleotide binding motif of hepatitis C virus NS4B can mediate cellular transformation and tumor formation without ha-ras co-transfection. Hepatology.

[12] Lee JM, Yang J, Newell P, Singh S, Parwani A, Friedman SL (2014). β-catenin signaling in hepatocellular cancer: implications in inflammation, fibrosis, and proliferation. Cancer Lett.

[13] Vilchez V, Turcios L, Marti F, Gedaly R (2016). Targeting Wnt/β-catenin pathway in hepatocellular carcinoma treatment. World J Gastroenterol.

[14] Fukutomi T, Zhou Y, Kawai S, Eguchi H, Wands JR, Li J (2005). Hepatitis C virus core protein stimulates hepatocyte growth: correlation with upregulation of wnt1 expression. Hepatology.

[15] Liu J, Ding X, Tang J, Cao Y, Hu P, Zhou F (2011). Enhancement of canonical Wnt/β-catenin signaling activity by HCV core protein promotes cell growth of hepatocellular carcinoma cells. PLoS One.

[16] Rogacki K, Kasprzak A, Stępiński A (2015). Alterations of Wnt/β-catenin signaling pathway in hepatocellular carcinomas associated with hepatitis C virus. Pol J Pathol.

[17] Jiang XH, Xie YT, Jiang B, Tang MY, Ma T, Peng H (2015). Inhibition of expression of hepatitis C virus 1b genotype core and NS4B genes in HepG2 cells using artificial microRNAs. Mol Med Rep.

[18] Guan CN, Chen XM, Lou HQ, Liao XH, Chen BY, Zhang PW (2012). Clinical significance of axin and β-catenin protein expression in primary hepatocellular carcinomas. Asian Pac J Cancer Prev.

[19] Kaur P, Mani S, Cros MP, Scoazec JY, Chemin I, Hainaut P (2012). Epigenetic silencing of sFRP1 activates the canonical Wnt pathway and contributes to increased cell growth and proliferation in hepatocellular carcinoma. Tumour Biol.

[20] Banerjee A, Ray RB, Ray R (2010). Oncogenic potential of hepatitis C virus proteins. Viruses.

[21] Liu J, Wang Z, Tang J, Tang R, Shan X, Zhang W (2011). Hepatitis C virus core protein activates Wnt/β-catenin signaling through multiple regulation of upstream molecules in the SMMC-7721 cell line. ArchVirol.

[22] Park CY, Choi SH, Kang SM, Kang JI, Ahn BY, Kim H (2009). Nonstructural 5A protein activates beta-catenin signaling cascades: implication of hepatitis C virus-induced liver pathogenesis. J Hepatol.

[23] Quan H, Zhou F, Nie D, Chen Q, Cai X, Shan X (2014). Hepatitis C virus core protein epigenetically silences SFRP1 and enhances HCC aggressiveness by inducing epithelial-mesenchymal transition. Oncogene.

[24] Huang S, Xie Y, Yang P, Chen P, Zhang L (2014). HCV core protein-induced down-regulation of microRNA-152 promoted aberrant proliferation by regulating Wnt1 in HepG2 cells. PLoS One.

[25] Zhang Y, Wei W, Cheng N, Wang K, Li B, Jiang X (2012). Hepatitis C virus-induced up-regulation of microRNA-155 promoteshepatocarcinogenesis by activating Wnt signaling. Hepatology.

[26] McGivern DR, Lemon SM (2011). Virus-specific mechanisms of carcinogenesis in hepatitis C virus associated liver cancer. Oncogene.

[27] Lemon SM, McGivern DR (2012). Is hepatitis C virus carcinogenic?. Gastroenterology.

[28] Rusyn I, Lemon SM (2014). Mechanisms of HCV-induced liver cancer: what did we learn from in vitro and animal studies?. Cancer Lett.

[29] Buck M (2008). Direct infection and replication of naturally occurring hepatitis C virus genotypes 1, 2, 3 and 4 in normal human hepatocyte cultures. PLoS One.

