# Supplementary material for: Effects of hepatitis C virus core protein and nonstructural protein 4B on the Wnt/β-catenin pathway
Source: BMC Microbiol. 2017 May 25;17:124. doi: 10.1186/s12866-017-1032-4 (PMC5445264; doi:10.1186/s12866-017-1032-4)
Supplement: Supplementary file 1 — Primers for PCR. (DOCX 17 kb) [file 12866_2017_1032_MOESM1_ESM.docx]

Additional file 1:Table S1.Primers for PCR.

Table S1 Primers for PCR

| Gene | Primer sequence (5’ to 3’ oriented) |
| --- | --- |
| *BamHI*-Core (forward) | CGCGGATCCGCCACCATGAGCACAAATCCTAAACCCCAAAGA |
| Core (reverse) | CTTAATCAGCTCGCTCACCATGGTGGCGGAAGCTGGGATGGTC |
| Core -mkate2 (forward) | GACCATCCCAGCTTCCGCCACCATGGTGAGCGAGCTGATTAAG |
| *BamHI*-mkate2 (forward) | CGCGGATCCGCCACCATGGTGAGCGAGCTGATTAAGGAGAAC |
| mkate2-*AscI*(reverse) | TAAGGCGCGCCTCATCTGTGCCCCAGTTTGCTAG |
| *BamHI*-NS4B (forward) | CGCGGATCCGCCACCATGGCCTCACACCTCCCTTACAT |
| NS4B (reverse) | CTTAATCAGCTCGCTCACCATGGTGCATGGCGTGGAGCAGTCCTC |
| NS4B-mkate2 (forward) | GAGGACTGCTCCACGCCATGCACCATGGTGAGCGAGCTGATTAAG |
