# Supplementary material for: Effects of hepatitis C virus core protein and nonstructural protein 4B on the Wnt/β-catenin pathway
Source: BMC Microbiol. 2017 May 25;17:124. doi: 10.1186/s12866-017-1032-4 (PMC5445264; doi:10.1186/s12866-017-1032-4)
Supplement: Supplementary file 2 — Sequencing results of the pLenti6.3-Core, pLenti6.3-NS4B and pLenti6.3-mkate2 (partial). (DOCX 274 kb) [file 12866_2017_1032_MOESM2_ESM.docx]

Additional file 2: Supporting Figure 2. Sequencing results of the pLenti6.3-Core, pLenti6.3-NS4B and pLenti6.3-mkate2.

A


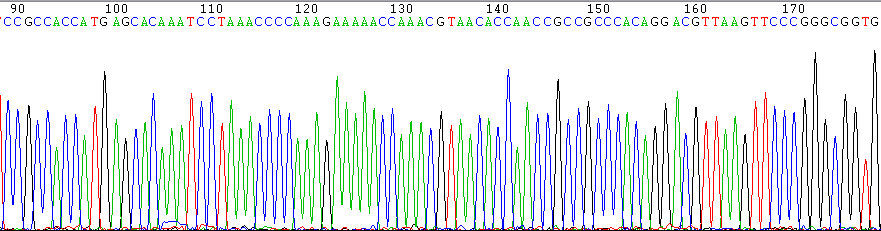


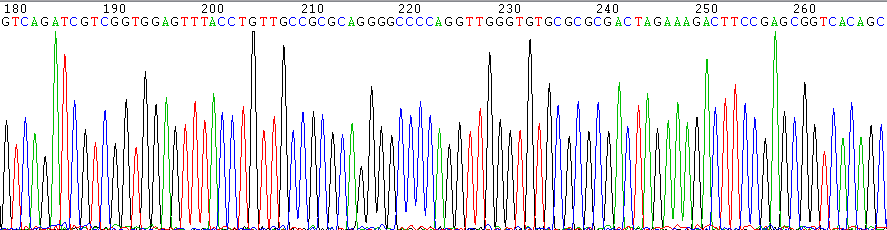


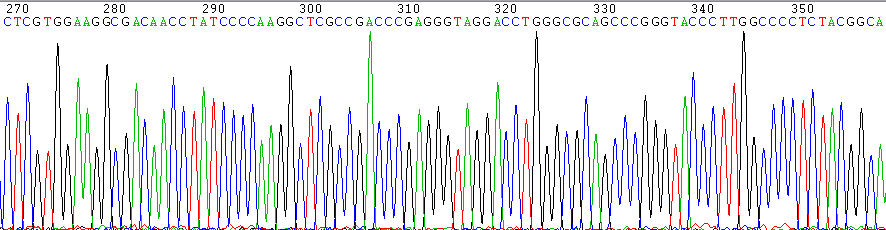


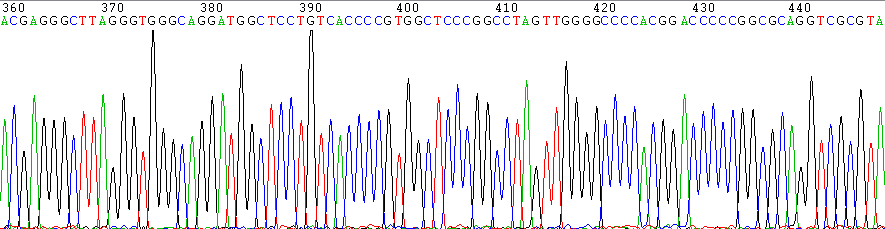


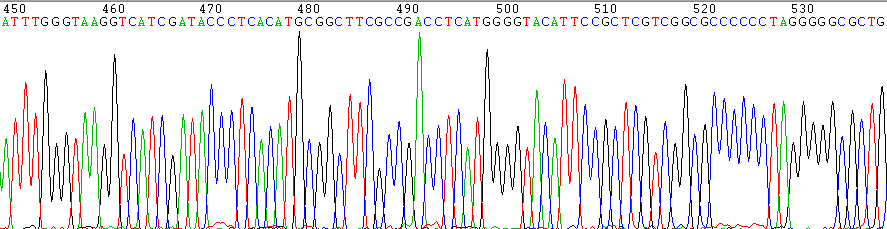


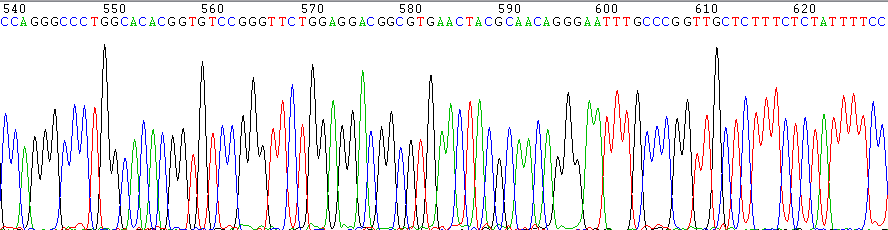


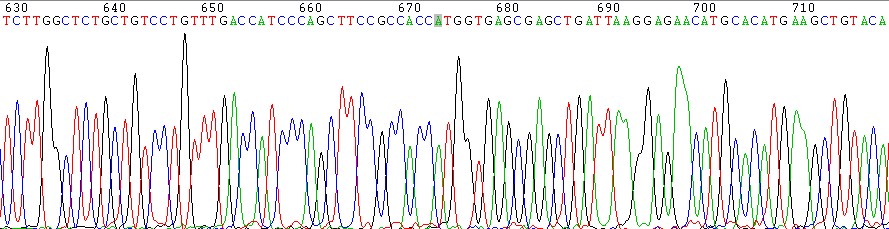


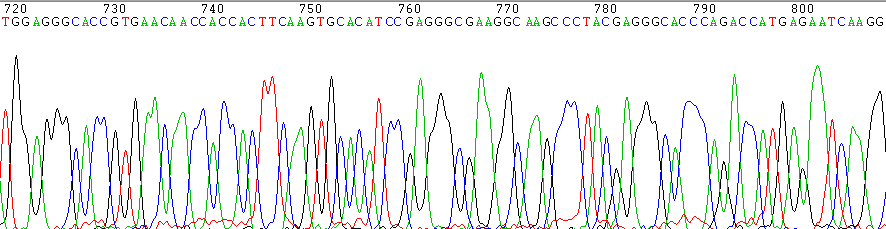


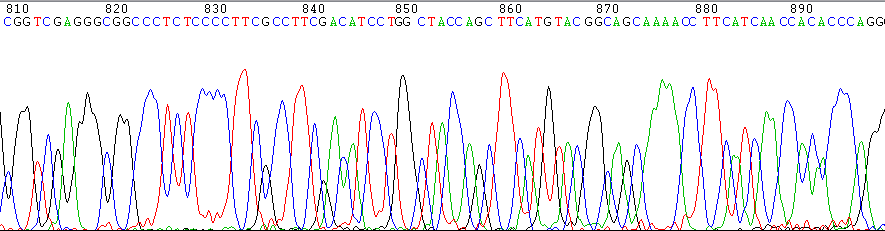


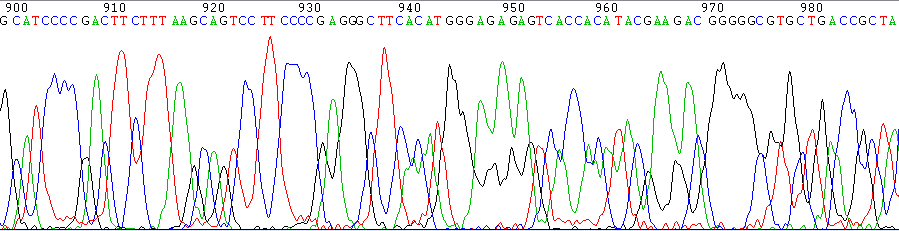

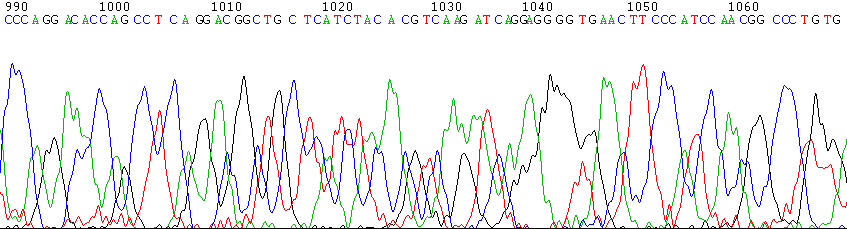


B


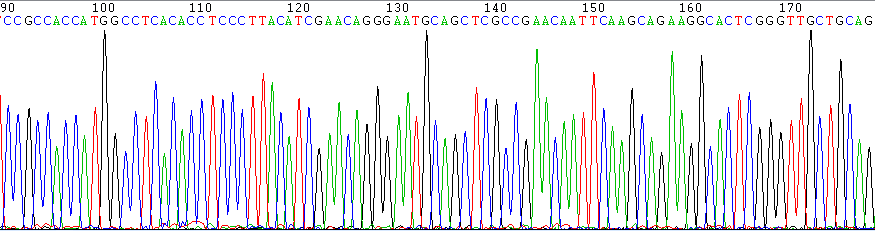


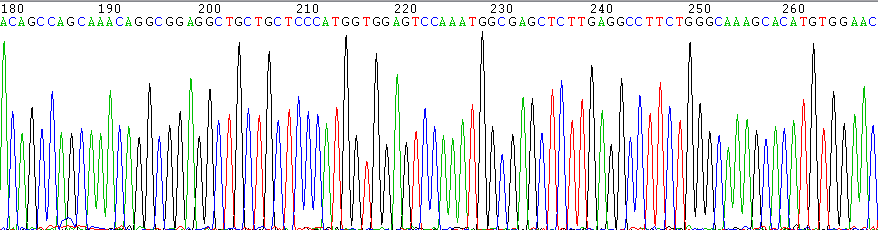


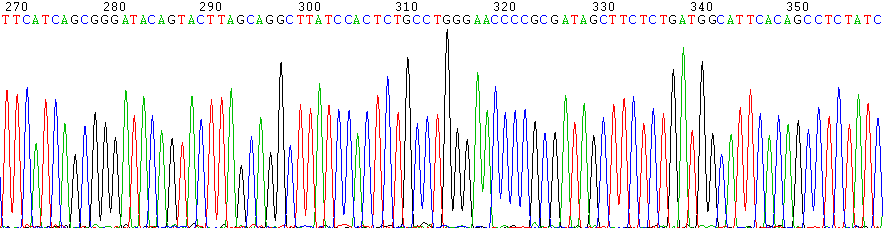


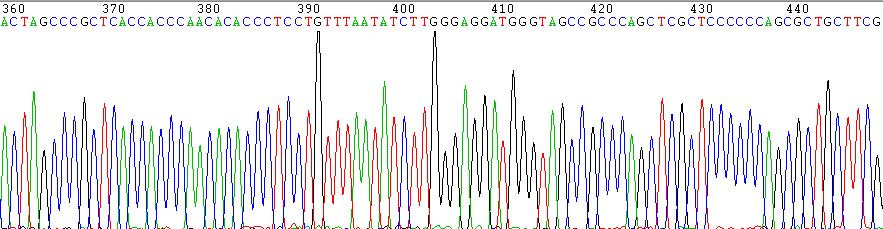


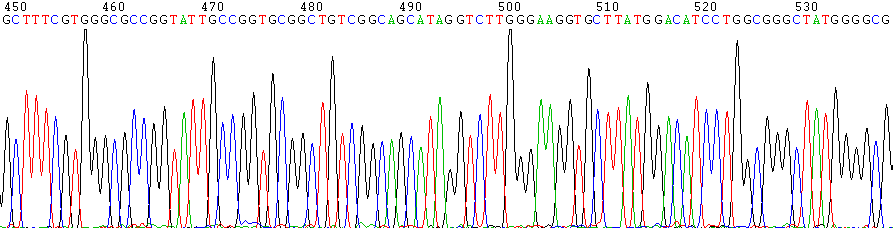


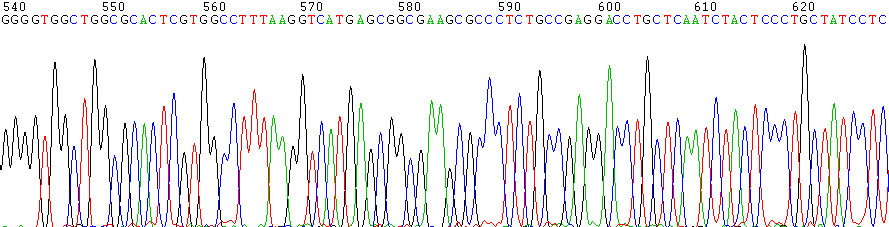


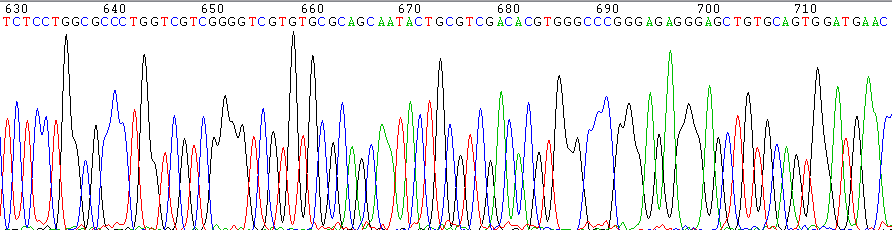


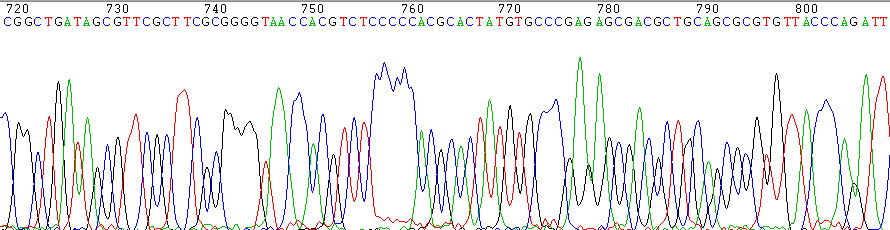


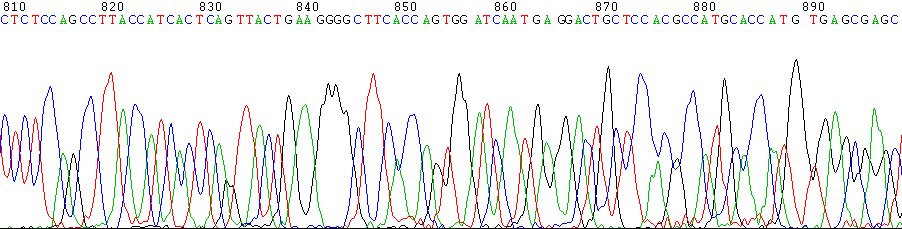


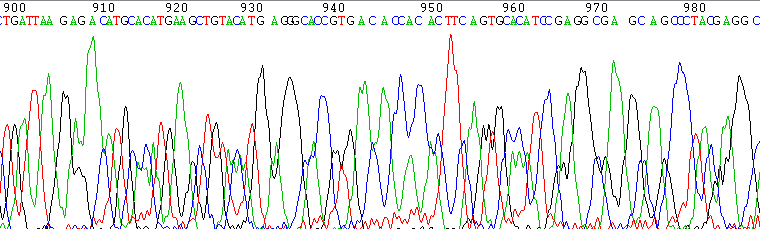


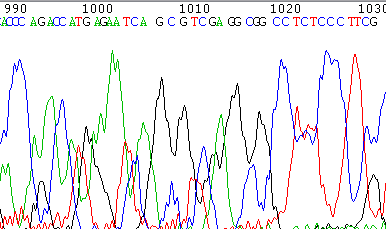


C


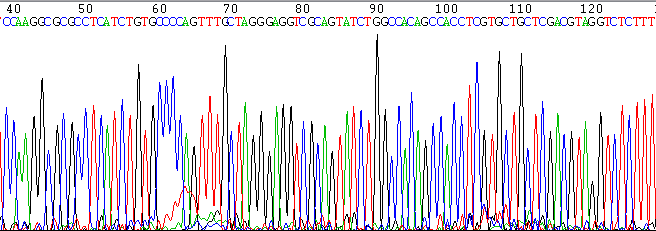


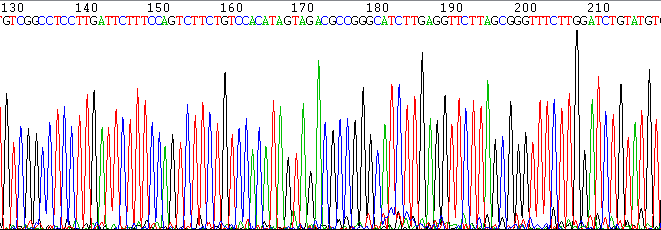


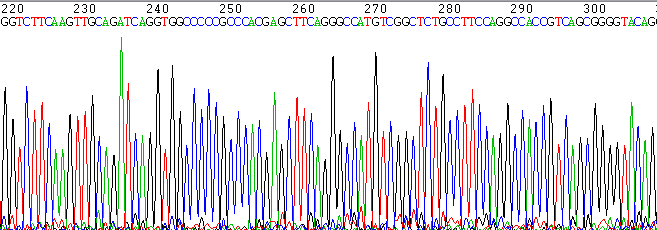


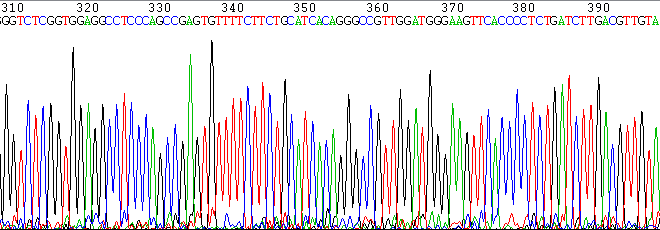


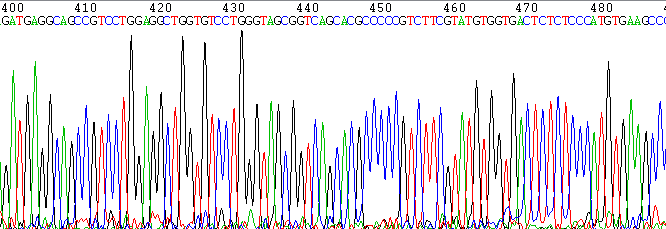


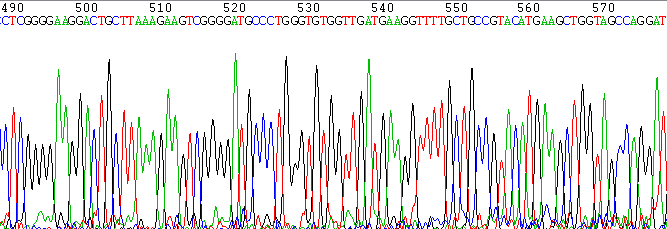


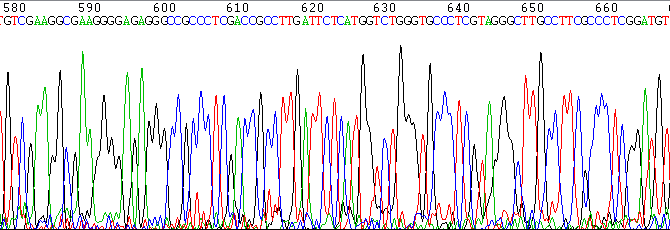


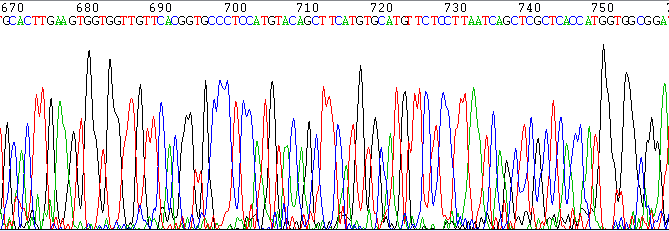


Supporting Figure 2. Sequencing results of the pLenti6.3-Core, pLenti6.3-NS4B and pLenti6.3-mkate2. A：Peak-figure of HCV Core gene sequencing. HCVCore 起始基因ATG位于nucleotide (nt) sequence 97-99位，终止基因GCC位于nt 667-669位，已用双下划线标出；B：Peak-figure of HCV NS4B gene sequencing.HCV-NS4B 起始基因ATG位于nt98-100位，终止基因TGC位于nt881-883位，已用双下划线标出；C：Peak-figure of mkate2 gene reversesequencing. mkate2 互补反向终止基因TCA位于nt51-53位，互补反向起始基因CAT位于nt747-749位，已用双下划线标出。
